# Association of vitamin D receptor TaqI and ApaI genetic polymorphisms with nephrolithiasis and end stage renal disease: a meta-analysis

**DOI:** 10.1186/s12881-019-0932-6

**Published:** 2019-12-10

**Authors:** Tajamul Hussain, Shaik M. Naushad, Anwar Ahmed, Salman Alamery, Arif A. Mohammed, Mohamed O. Abdelkader, Nasser Abobakr Nasser Alkhrm

**Affiliations:** 1 Center of Excellence in Biotechnology Research, Department of Biochemistry, College of Science Building 5, King Saud University, Riyadh, 11451 Saudi Arabia; 2 Biochemical Genetics, Sandor Life Sciences Pvt. Ltd, Hyderabad, India; 3 Department of Biochemistry, College of Science, King Saud University, Riyadh, Saudi Arabia

**Keywords:** Vitamin D receptor gene polymorphism, End stage renal disease, Nephrolithiasis, Diabetic nephropathy, Meta-analysis

## Abstract

**Background:**

The deficiency of vitamin D receptor (VDR) or its ligand, vitamin D3, is linked to the development of renal diseases. The TaqI (rs731236) and ApaI (rs7975232) polymorphisms of VDR gene are widely studied for their association with renal disease risk. However, studies have largely been ambiguous.

**Methods:**

Meta-analysis was carried out to clarify the association of TaqI (2777 cases and 3522 controls) and ApaI (2440 cases and 3279 controls) polymorphisms with nephrolithiasis (NL), diabetic nephropathy (DN) and end stage renal disease (ESRD).

**Results:**

The VDR TaqI C-allele under allele contrast was significantly associated with ESRD in both fixed effect and random effect models, and ApaI C-allele with ESRD only under fixed effect model. Cochrane Q-test showed no evidence of heterogeneity for TaqI polymorphism and a significant heterogeneity for Apa I polymorphism. No publication bias was observed for both the polymorphisms.

**Conclusions:**

The present meta-analysis identifies TaqI and ApaI polymorphisms of VDR gene as risk factors for renal diseases.

## Introduction

In human skin, solar rays facilitate the formation of vitamin D3 from 7-dehydrocholesterol. The vitamin D3 undergoes two-step hydroxylation to form 25-hydroxy vitamin D3 (25-OHD3) and biologically active 1,25-dihydroxyvitamin D3 (1,25-(OH)_2_D3) [1]. Vitamin D receptor (VDR) is a ligand-activated transcriptional factor requiring 1,25(OH)2D for its activation [2]. The deficiency of 25OHD or VDR is reported to activate renin-angiotensin system resulting in high angiotensin II levels, which damage renal parenchyma leading to increased risk for renal disease [3]. Considering the pivotal role of VDR in maintaining normal renal function, a number of studies have explored the possibility of association of VDR gene polymorphisms with renal disease risk. Among VDR polymorphisms reported to date, ApaI, and TaqI are widely studied for their association with ESRD, NL and DN [4–6]. The ApaI variant (rs7975232), which results in A to C transition, is located in the intron 8 of VDR gene, while TaqI variant (rs731236), which results in T to C transition is located in exon 9 [7].

The rs7975232 (NG_008731.1:g.64978G > T) is an intronic variant predicted to influence splice site changes that might affect the translation of VDR. The frequency of this variant is high as evidenced by 734 and 16,751 homozygous mutants in 1000G and ExAC databases. The rs731236 (NG_008731.1:g.65058 T > C) variant is near the exon-intron boundary (GCTG/attg) and hence likely to influence splicing and thus might affect the translation of VDR. The frequency of this variant is lower than that of rs7975232 with 242 and 7505 homozygous mutants identified in 1000G and ExAC databases.

Importantly, genetic studies examining the role of TakI and ApaI polymorphisms in the pathogeneses of NL, DN and ESRD remained ambiguous [4–6, 8–12]. Considering the significance of VDR signaling in the protection against renal diseases and the ambiguity in the studies relating VDR gene polymorphism with the disease etiology, present meta-analysis comprising 2669 renal disease cases and 3342 controls was carried out to clarify the association of VDR gene TaqI and ApaI polymorphisms with nephrolithiasis, ESRD and diabetic nephropathy. Upon reviewer's suggestion the data related this sentence has been removed from the manuscript, regrettably we failed to delete this sentence in our revised submission.

## Methods

### Data extraction

The literature retrieval was carried out using keywords: vitamin D receptor or VDR, renal disease, nephrolithiasis or urolithiasis, diabetic nephropathy, TaqI (rs731236) and ApaI (rs7975232) in PubMed, Medline and google scholar databases. All the free full texts were retrieved and wherever full text was not available, reprint request was sent to the corresponding author of the respective article. The criteria to include in the meta-analysis were: 1) availability of full text of the article, 2) inclusion of studies involving both cases and controls (either online or through reprint from the corresponding author), 3) availability of raw data on genotypes, and 4) restricting to studies published in only English language. The information related to each study such as first author, year of study, ethnic group or population studied, distribution of genotypes in cases and controls etc. was computed. The decision on the studies to be included in meta-analysis was taken by all the authors of this study.

### Meta-analysis

The data computed in four columns wherein first two columns represent the number of variant alleles in cases and controls and last two columns represent the number of ancestral alleles in cases and controls. Log (odds ratio) or effect size and standard error (SE) are calculated based on these four column data. Based on these two parameters, variance (SE^2^), weight and 95% confidence interval of effect size were calculated. Cochrane Q test and I^2^ statistics were performed to test the heterogeneity in the association. The plot of 1/SE and Z-statistics was also used as an index to test heterogeneity. The publication bias was based on the rank correlation of SE and v. The fixed effect and random effect models were generated based on Mantel Haenszel and DerSimonian Lair’s methods, respectively. If no evidence of heterogeneity was found, fixed effect model was considered. If test heterogeneity was significant, random effect model was considered.

## Results

**Fig. 1:**
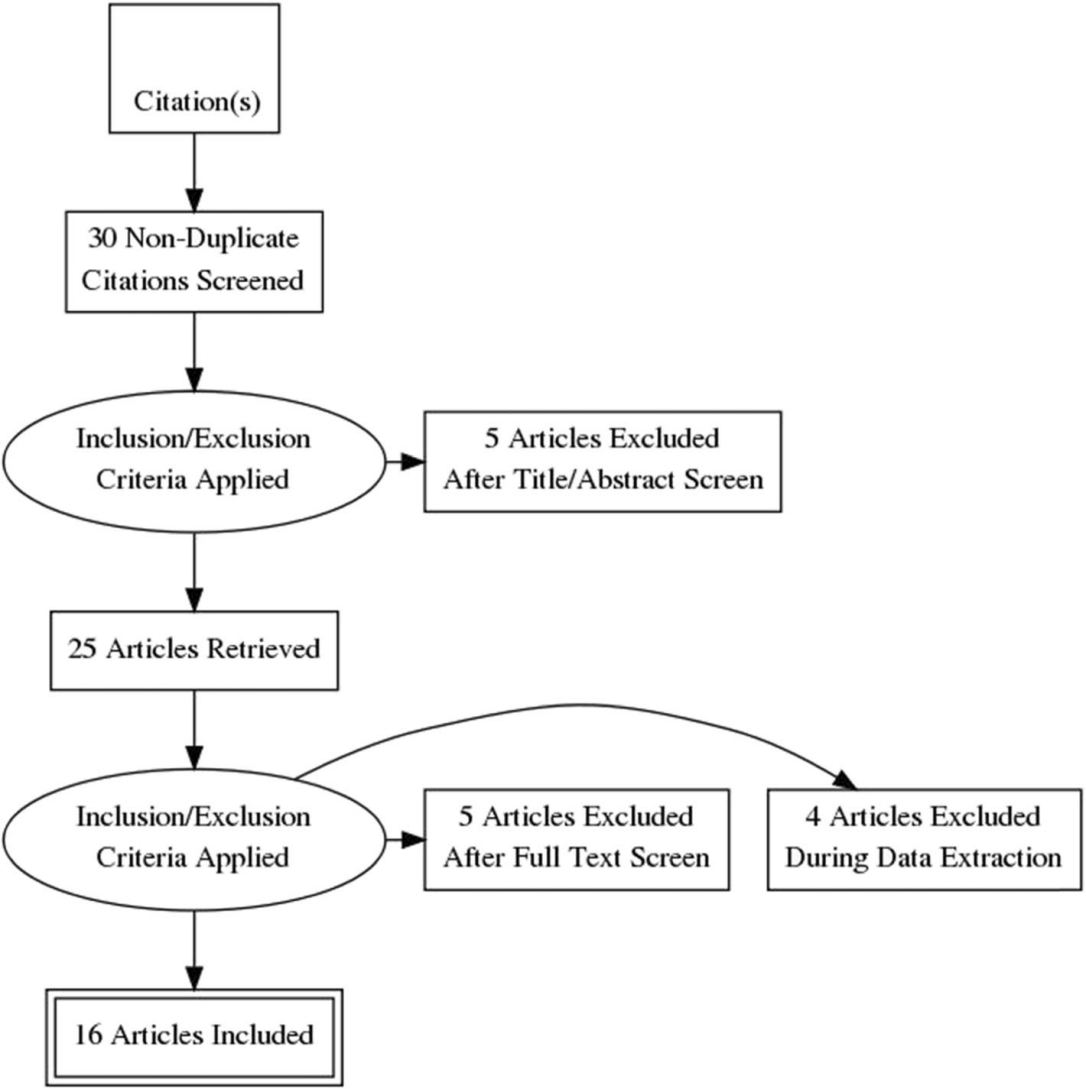
PRISMA flowchart showing the steps in meta-analysis data extraction

Figure 1 depicts the data extraction process for the meta-analysis. Of the 16 case-control studies retrieved on the association of TaqI polymorphism with renal disease (Table 1), four studies showed deviation from Hardy-Weinberg equilibrium [7, 13–15]. Among the different population groups included in this meta-analysis, the largest being that of Turkish representing five case-control studies [16–20], two studies from India [21, 22] and one each from China [23], Ireland [24], Italy [25], Spain [26] and Croatia [27]. In total, the final meta-analysis was based on the data of 2777 cases and 3522 controls representing 16 case-control studies.

**Table 1:** Distribution of VDR1 TaqI polymorphism in different case-control studies

Author	Year	Country	Renal disease type	Genotypes						C-allele frequency	
				Cases			Control				
				TT	TC	CC	TT	TC	CC	Cases	Control
Wang [23]	2016	China	ESRD	215	197	40	474	358	72	0.31	0.28
Cakir [20]	2016	Turkey	NL	35	44	19	31	29	10	0.42	0.35
Guha [13]	2015	India	NL	58	82	60	65	58	77	0.51	0.53
Martin [24]	2010	Ireland	DN	225	327	103	249	327	98	0.41	0.39
Ozkaya [16]	2003	Turkey	NL	33	27	4	50	30	10	0.27	0.28
Mossetti [25]	2003	Italy	NL	80	104	36	35	66	13	0.40	0.40
Bucan [27]	2009	Croatia	DN	5	6	3	13	14	6	0.43	0.39
Nosratabadi [7]	2010	Iran	DN	9	55	36	4	63	33	0.64	0.65
Goknar [15]	2016	Turkey	NL	25	41	12	14	43	3	0.42	0.41
Tripathi [21]	2010	India	ESRD	105	115	38	267	228	74	0.37	0.33
Mittal [22]	2010	India	NL	56	61	8	84	50	16	0.31	0.27
Moyano [26]	2007	Spain	NL	15	23	13	9	11	1	0.48	0.31
Gunes [17]	2006	Turkey	NL	37	63	10	61	73	16	0.38	0.35
Seyhan [18]	2007	Turkey	NL	27	35	18	13	25	2	0.44	0.36
Aykan [19]	2015	Turkey	NL	67	61	36	66	86	15	0.41	0.35
Han [14]	2015	China	NL	102	6	0	160	16	4	0.03	0.07

The following studies were shown to have deviation from HWE: Guha et al. (*p* < 0.0001), Nosratabadi et al. (*p* = 0.0008), Goknar et al. (*p* = 0.0008) and Han et al. (p = 0.0008)

*ESRD* end stage renal disease, *NL* nephrolithiasis, *DN* diabetic nephropathy

**Fig. 2:**
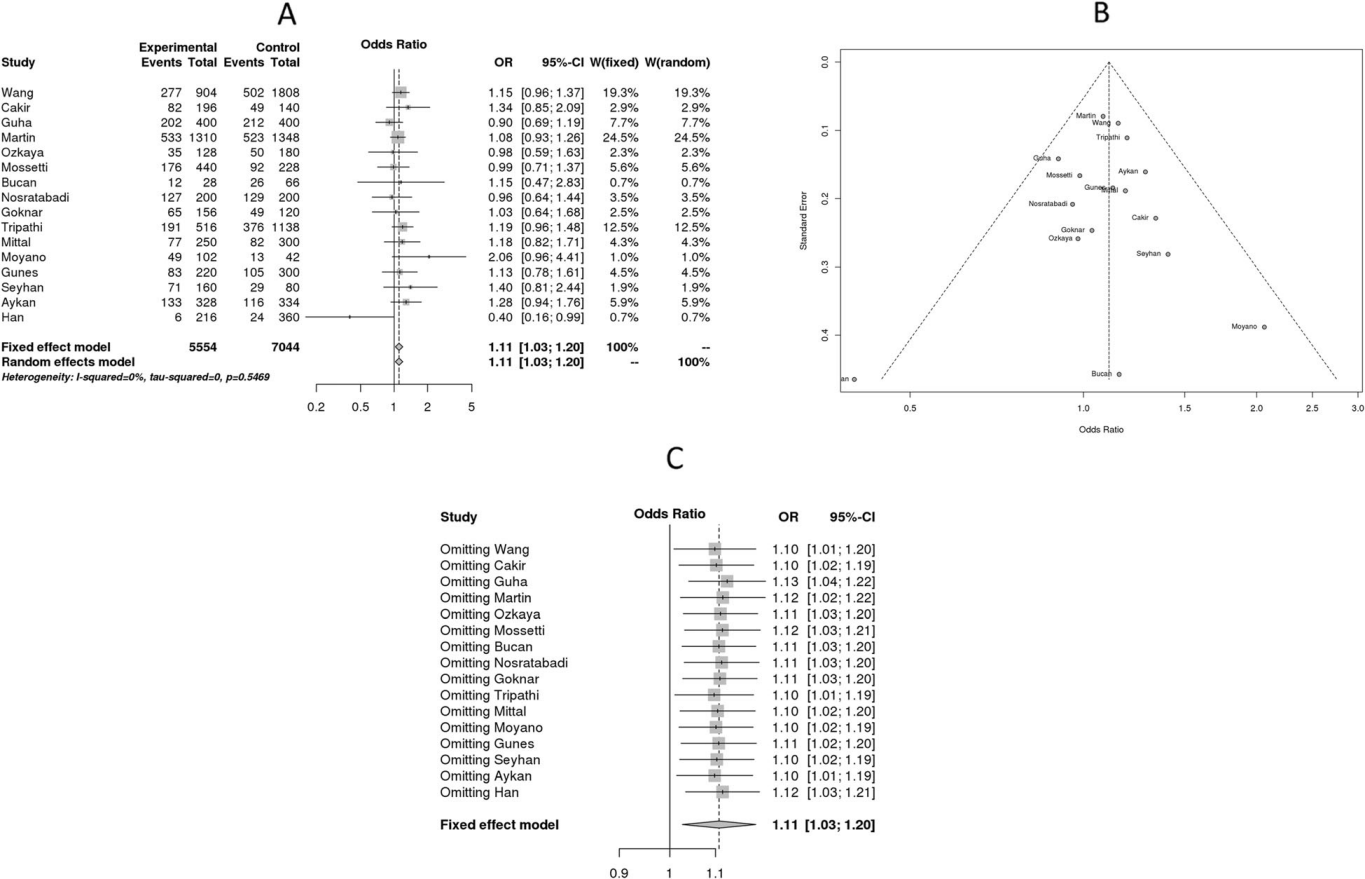
Meta-analysis of association studies on VDR TaqI polymorphism vs. risk for renal disease. Forest plot: The terms experimental and control groups corresponds to cases and controls. Number of variant alleles was considered as events with respect to total number of alleles tested. This meta-analysis was based on 16 case-control studies representing seven population groups. VDR TaqI polymorphism was shown to exert risk for renal disease both in fixed effect and random effect models. Funnel Diagram: It is depicting that no heterogeneity in association. Sensitivity analysis: Exclusion of any of the study is not influencing the result

Cochrane Q-test (Q: 13.72, *p* = 0.54) and I^2^ (0.00) statistics showed no evidence of heterogeneity in association. Egger’s test revealed no evidence of publication bias (*p* = 0.14). The VDR TaqI C-allele, under allele contrast fixed effect model, was associated with renal diseases calculated collectively for DN, ESRD and NL (OR: 1.11, 95% CI: 1.03–1.20, *p* = 0.008). (Figure 2) As shown Table 2, subtype analysis revealed Taql C- allele to be associated with ESRD (OR: 1.17, 95% CI: 1.02–1.34, *p* = 0.03) (Fig. 2). Among the different ethnic groups, Turkish population showed strong association between VDR TaqI polymorphism and renal disease in allele contrast model (C vs. T, OR: 1.19, 95% CI: 1.01–1.42, *p* = 0.04). Sensitivity analysis revealed that omitting either of the studies had no effect on overall outcome of disease risk.

**Table 2:** Subgroup analysis showing disease-specific risk with VDR TaqI polymorphism

Model	Type of disease	N	OR	95% CI	*P* value
Allele contrast (A vs. a)	Overall	16	1.11	[1.0262; 1.1967]	0.009
	ESRD	2	1.17	[1.0171; 1.3357]	0.028
	NL	11	1.09	[0.9673; 1.2356]	0.153
	DN	3	1.07	[0.9250; 1.2322]	0.371
Recessive model (AA vs. Aa+aa)	Overall	16	1.19	[0.9266; 1.5392]	0.170
	ESRD	2	1.14	[0.8497; 1.5235]	0.386
	NL	11	1.32	[0.8084; 2.1503]	0.268
	DN	3	1.11	[0.8527; 1.4432]	0.439
Dominant model (AA+Aa vs. aa)	Overall	16	1.14	[1.0234; 1.2709]	0.017
	ESRD	2	1.24	[1.0367; 1.4863]	0.019
	NL	11	1.09	[0.9148; 1.2930]	0.342
	DN	3	1.09	[0.8737; 1.3505]	0.456
Overdominant (Aa vs. AA + aa)	Overall	16	0.99	[0.8106; 1.2040]	0.904
	ESRD	2	1.19	[0.9904; 1.4233]	0.063
	NL	11	0.92	[0.6575; 1.2975]	0.647
	DN	3	1.01	[0.8261; 1.2289]	0.940
pairw1 (AA vs. aa)	Overall	16	1.20	[1.0117; 1.4232]	0.036
	ESRD	2	1.26	[0.9280; 1.7151]	0.138
	NL	11	1.23	[0.9346; 1.6077]	0.141
	DN	3	1.11	[0.8081; 1.5149]	0.528
pairw2 (AA vs. Aa)	Overall	16	1.16	[0.8525; 1.5857]	0.341
	ESRD	2	1.01	[0.7443; 1.3803]	0.932
	NL	11	1.30	[0.7200; 2.3483]	0.384
	DN	3	1.09	[0.8304; 1.4407]	0.524
pairw3 (Aa vs. aa)	Overall	16	1.09	[0.9167; 1.2888]	0.337
	ESRD	2	1.24	[1.0233; 1.4966]	0.028
	NL	11	1.04	[0.7873; 1.3666]	0.795
	DN	3	1.07	[0.8487; 1.3425]	0.577

**Fig. 3:**
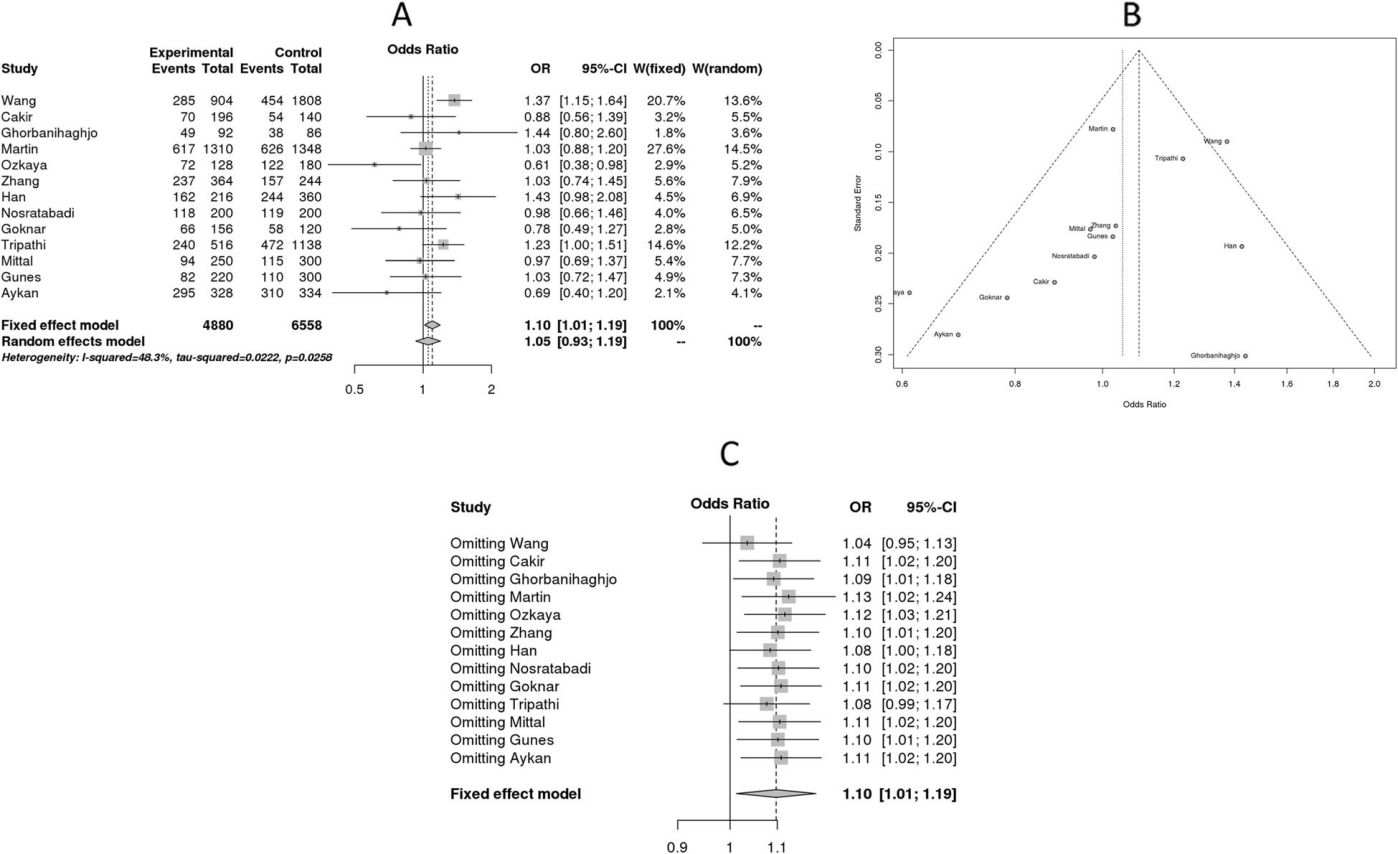
Meta-analysis of association studies on VDR ApaI polymorphism vs. risk for renal disease. Forest plot: The terms experimental and control groups correspond to cases and controls. Number of variant alleles were considered as events with respect to total number of alleles tested. This meta-analysis was based on 13 case-control studies representing 5 population groups. VDR ApaI polymorphism was shown to exert risk for renal disease only in fixed effect model, but not in random effect model. Funnel Diagram: It is depicting that two studies are contributing to heterogeneity. Sensitivity analysis: Excluding two studies is influencing the results

Of the 13 case-control studies (2440 cases and 3279 controls) retrieved on the association of ApaI polymorphism with renal disease (Table 3), five studies deviated from Hardy-Weinberg equilibrium [7, 15, 19, 21, 28]. Among the studies in accordance with HWE equilibrium, 3 studies were from Turkey [16, 17, 20], two from China [14, 23], and one each from Ireland [24] and Iran [29]. Cochrane Q-test (Q: 17.01, *p* = 0.03) and I^2^ (48.3) statistics showed high-degree of heterogeneity in association. Egger’s test revealed no evidence of publication bias (*p* = 0.54). The fixed effect model showed positive association of VDR ApaI polymorphism with all the renal disease cases (C vs. A, OR: 1.10, 95% CI: 1.01–1.19), whereas, random effect model showed null association (OR: 1.05, 95% CI: 0.93–1.19) (Fig. 3). Sensitivity analysis for ApaI polymorphism revealed that the sources of heterogeneity are two studies i.e. Wang et al. and Tripathi et al. However, overall trend suggests ApaI variant as a risk factor for renal disease. As shown in Table 4, subgroup analysis revealed association of VDR ApaI polymorphism with ESRD (C vs. A, OR: 1.31, 95% CI: 1.15–1.50, *p* = 0.0001) and no association with NL and DN.

**Table 3:** Distribution of VDR1 ApaI polymorphism across different case-controls studies

Author	Year	Country	Renal disease type	Genotypes						C-allele frequency	
				Cases			Control				
				AA	AC	CC	AA	AC	CC	Cases	Controls
Wang [23]	2016	China	ESRD	206	207	39	502	350	52	0.32	0.25
Cakir [20]	2016	Turkey	NL	43	40	15	26	34	10	0.36	0.39
Ghorbanihaghjo [29]	2014	Iran	CH	10	23	13	16	16	11	0.53	0.44
Martin [24]	2010	Ireland	DN	185	323	147	200	322	152	0.47	0.46
Ozkaya [16]	2003	Turkey	NL	13	30	21	4	50	36	0.56	0.68
Zhang [28]	2012	China	DN	19	89	74	11	65	46	0.65	0.64
Han [14]	2015	China	DN	2	50	56	18	80	82	0.75	0.68
Nosratabadi [7]	2010	Iran	DN	9	64	27	9	63	28	0.59	0.60
Goknar [15]	2016	Turkey	NL	24	42	12	11	40	9	0.42	0.48
Tripathi [21]	2010	India	ESRD	80	116	62	171	324	74	0.47	0.41
Mittal [22]	2010	India	NL	43	70	12	57	71	22	0.38	0.38
Gunes [17]	2006	Turkey	NL	40	58	12	59	72	19	0.37	0.37
Aykan [19]	2015	Turkey	NL	14	5	145	12	0	155	0.90	0.93

The following studies were shown to have deviation from HWE: Ozkaya et al. (p = 0.03), Nosratabadi et al. (*p* = 0.009), Goknar et al. (*p* = 0.03), Tripathi et al. (p < 0.0001) and Aykan et al. (*p* < 0.0001)

*ESRD* end stage renal disease, *NL* nephrolithiasis, *CH* chronic hemodialysis, *DN* diabetic nephropathy

**Table 4:** Subgroup analysis showing disease-specific risk with VDR ApaI polymorphism

Model	Type of disease	N	OR	95% CI	p-val
Allele contrast (A vs. a)	Overall	13	1.05	[0.9282; 1.1931]	0.4259
	ESRD	2	1.31	[1.1454; 1.4996]	0.0001
	NL	6	0.86	[0.7193; 1.0175]	0.0777
	CH	1	1.44	[0.7974; 2.5983]	0.2268
	DN	4	1.06	[0.9361; 1.1997]	0.3589
Recessive model (AA vs. Aa+aa)	Overall	13	1.10	[0.8891; 1.3548]	0.3865
	ESRD	2	1.85	[1.3925; 2.4544]	0.0000
	NL	6	0.77	[0.5591; 1.0553]	0.1035
	CH	1	1.15	[0.4482; 2.9300]	0.7760
	DN	4	1.06	[0.8695; 1.2818]	0.5840
Dominant model (AA+Aa vs. aa)	Overall	13	1.03	[0.8131; 1.3008]	0.8153
	ESRD	2	1.21	[0.7844; 1.8716]	0.3868
	NL	6	0.76	[0.5034; 1.1586]	0.2049
	CH	1	2.13	[0.8380; 5.4311]	0.1120
	DN	4	1.09	[0.8749; 1.3545]	0.4466
Overdominant (Aa vs. AA + aa)	Overall	13	0.99	[0.8143; 1.2066]	0.9300
	ESRD	2	0.91	[0.4290; 1.9490]	0.8167
	NL	6	0.96	[0.6559; 1.3933]	0.8147
	CH	1	1.69	[0.7239; 3.9340]	0.2256
	DN	4	1.03	[0.8660; 1.2221]	0.7472
pairw1 (AA vs. aa)	Overall	13	1.09	[0.8006; 1.4779]	0.5907
	ESRD	2	1.81	[1.3275; 2.4638]	0.0002
	NL	6	0.70	[0.4803; 1.0158]	0.0604
	CH	1	1.89	[0.6130; 5.8330]	0.2677
	DN	4	1.09	[0.8307; 1.4252]	0.5399
pairw2 (AA vs. Aa)	Overall	13	1.10	[0.8709; 1.3854]	0.4280
	ESRD	2	1.74	[0.9540; 3.1683]	0.0709
	NL	6	0.86	[0.5968; 1.2327]	0.4068
	CH	1	0.82	[0.2948; 2.2927]	0.7082
	DN	4	1.02	[0.8306; 1.2477]	0.8635
pairw3 (Aa vs. aa)	Overall	13	1.03	[0.7832; 1.3445]	0.8515
	ESRD	2	1.06	[0.5720; 1.9761]	0.8464
	NL	6	0.79	[0.4507; 1.3857]	0.4113
	CH	1	2.30	[0.8331; 6.3500]	0.1080
	DN	4	1.10	[0.8688; 1.3802]	0.4417

## Discussion

Deficiency of vitamin D or defective activation of VDR by its ligand, 1,25-dihydroxy vitamin D results in secondary hyperparathyroidism, angiotensin II-mediated renal damage and renal disease pathogenesis [3]. On the other hand, VDR activation suppressed inflammatory cell infiltration and inhibited nuclear factor-κB activation [30]. Likewise, active vitamin D3 and lentivirus-mediated transforming growth factor-β (TGF-β) interference effectively reduced renal fibrosis in rat models [31]. These observations highlight the importance of VDR signaling in maintaining normal renal function. Accordingly, a number of studies have investigated the effects of polymorphisms in VDR gene on renal disease etiology. Among these, TaqI, and ApaI polymorphisms are widely studied [4–6]. However, there is a considerable ambiguity among these genetic studies, possibly stemming from sample size, ethnicity or gene-environmental interactions [4–6, 8–12]. To clarify whether TaqI and apaI polymorphisms have a role in renal disease pathogenesis, this meta-analysis comprising 2777 renal disease cases including DN, NL and ESRD and 3522 healthy controls was carried out. The present meta-analysis revealed an increased disease risk for subjects harboring TaqI C-allele under fixed and random effect models. Subgroup analysis based on type of renal disease showed that VDR TaqI polymorphism is associated with ESRD in allele contrast model, whereas no significant association was found between TaqI polymorphism and DN and NL. In the case of ApaI polymorphism, Apal C-allele was found to be linked to ESRD, but not with DM or NL under fixed effect model. Earlier, Yang et al. performed a meta-analysis on 1510 cases and 1812 controls and found no association of BsmI, FokI, TaqI, and ApaI polymorphisms of VDR with end-stage renal disease. Inclusion of more studies benefited the current meta-analysis.

The direct role of solar rays in the synthesis of vitamin D is well known. In human skin, solar rays facilitate the formation of vitamin D3 from 7-dehydrocholesterol, which is evident from the presence of higher mean serum vitamin D levels in summer than in winter [32]. Likewise, higher vitamin D levels were found in populations living in regions known to have longer durations of sun exposure [33].

## Conclusions

This meta-analysis revealed the association of VDR TaqI and ApaI polymorphisms with ESRD risk. This is the first meta-analysis study to simultaneously evaluate the association of DN, NL and ESRD with renal disease risk. Ethnicity, sample size, gene-environmental interactions appear to be responsible for inconsistencies observed in the association studies examining VDR polymorphisms and renal diseases. The limitations of this meta-analysis include; exclusion of studies where raw data or full text were not accessible and one-to-one correlation between vitamin D3 profile and risk could not be established as no parallel studies were conducted.

## Data Availability

All data generated or analyzed during this study are included in this manuscript.

## Abbreviations

1,25 (OH)_2_D3: 1,25-dihydroxyvitamin D3
25-OHD3: 25-hydroxy vitamin D3
DN: diabetic nephropathy
ESRD: end stage renal disease
NL: nephrolithiasis
VDR: vitamin D receptor
